# A survey of innovation management among persons with disabilities in Malaysia

**DOI:** 10.12688/f1000research.74202.1

**Published:** 2022-02-14

**Authors:** Yuen Yee Yen, Eddie Wu Jian Yong, Wendy Teoh Ming Yen

**Affiliations:** 1 Faculty of Business, Multimedia University, Jalan Ayer Keroh Lama, Bukit Beruang, 75450, Malaysia

**Keywords:** Innovation Management, Disability Management, Innovation Barriers, Involvement Motivator, Inclusive Society

## Abstract

Background: As Malaysia struggles with the battle to retain talented workers, the retention of persons with disabilities (PWDs) remains a major challenge in innovation management. Malaysia currently has the lowest retention of PWDs in innovation management in The Association of Southeast Asian Nations (ASEAN). The purpose of this study is to develop a unique framework to enhance the inclusion of PWDs in Malaysia.

Methods: A questionnaire was distributed to 200 PWDs in the central region of Malaysia.

Results: Based on the results of this research, four crucial variables (salary, compensation, career advancement and reward management) contributed to the lack of involvement in innovation management among PWDs in Malaysia.

Conclusions: This study only focuses on 200 PWDs in Malaysia. Despite the fact that PWDs’ involvement in innovation management is the lowest, there is a lack of research initiative and practitioner commitment to address this issue. Serving as preliminary research in Malaysia, this study develops a unique framework to fill the gap.

## Introduction

As Malaysia struggles with the battle for talented employees following the COVID-19 pandemic, retaining skilled persons with disabilities (PWDs) remains a major challenge. PWD refers to those who have an impairment of the body. hearing or speech that makes it more difficult to perform certain activities alone (activity limitation) and interact with people around them (participation restrictions). Innovation management involvement refers to the participation of PWDs in contributing or managing ideas that result in the introduction of new goods or services or improvement in existing goods or services. It is challenging to retain PWDs working in the field of innovation management because PWDs have a high tendency to leave the workforce due to physical conditions and the facilitating conditions of the working environment. Malaysia’s PWDs retention in the field of innovation management remains low at 12.6 percent in 2020, ( Ta, Wah & Leng, 2021). In the global competitive world, PWDs have high potential to be excellent product and service innovators and help organizations lead with core competencies if they are given equal employment opportunities as normal workers. As Boehm *et al.* (2020) argues, PWDs are more capable of producing and generating innovative ideas compared to workers without disabilities as PWDs are always more aware of changes in the work environment.

According to Brown (2018), 82% of PWDs do not often participate in innovation management for a period of more than two years. This is a significant barrier to organizations, which hinders the business’ ability to maintain a healthy product innovation life cycle. Employers do not have sufficient knowledge or the competence to identify and utilize the innovation capability of PWDs in order to enhance PWDs’ job satisfaction and reduce their turnover. High rates of PWDs’ turnover cause high cost in innovation management for the company. The company will face great difficulties in achieving the business innovation goal and commercializing the innovation in the market.

The involvement of PWDs in innovation management in Malaysia has not yet reached the 40% global standard, which is far behind other countries in Asia ( Meeks *et al.*, 2019). Many organizations in Malaysia are unable to meet the needs of PWDs in terms of better career opportunities, lucrative salary, better working environments and rewards ( Ta *et al*., 2021). Companies should consider the retention of PWDs as a critical issue in order to save employers time and money to find replacement workers. However, most of the studies on disability management focus on countries such as India ( Nautiyal, 2021), Australia ( Kilpatrick *et al*., 2017) and Africa ( Vincent, & Chiwandire, 2019). Little attention has been given to enhancing innovation management of PWDs in Malaysia.

Despite the fact that PWDs’ involvement in innovation management is the lowest, there is a lack of research initiative and practitioner commitment to address this issue. Serving as preliminary research in Malaysia, this study develops a unique framework to fill the gap. The main purpose of this research is to examine PWDs’ innovation management involvement by taking into consideration factors such as risk-taking, reward management, job satisfaction, salary, career advancement and compensation.

## Literature review

Innovation management involvement refers to PWDs’ willingness to engage in product and service innovation ( Park & Park, 2019).

### Risk-taking

Risk-taking is one of the key determinants of PWDs involvement in innovation management. It examines whether PWDs are willing to take bold actions into new product and service innovation by investing their efforts with uncertain outcomes ( Suresh & Dyaram, 2020). Risk-taking behaviors shape and motivate the potential of PWDs to improve capability to participate in and achieve greater satisfaction through innovation management ( McKenzie, 2021).

### Job satisfaction

Job satisfaction is another factor that can determine innovation management involvement. Employees can consider their level of job satisfaction to determine whether they like or dislike their product and service innovation jobs ( Brown, 2019). The lower job satisfaction of PWDs can reflect their negative innovation interest and lack of involvement in contributing new ideas to the organization ( Boehm *et al*., 2020). Job satisfaction could be measured by the amount of related stress, and hours spent in innovation ( Kilpatrick *et al*., 2017). The higher an employee’s job satisfaction, the longer the hours spent in innovation.

### Reward

Reward refers to how an organization manages equitable monetary and non-monetary incentives in order to encourage PWDs to contribute their original ideas for improving the existing product and service ( McKenzie, 2021). It includes analyzing and managing PWDs’ welfare through structured procedures based on their innovation outcomes ( Baser *et al*., 2017). Sufficient reward makes PWDs feel their ideas are appreciated within the company ( Meeks *et al*., 2019). Organizations can express their gratitude through rewards such as gifts, company trips or offering extra leave.

### Salary

Salary is another factor that encourages PWDs to work hard in innovation and to accelerate self-worth in the company. Salary is determined by comparing market and area pay rates for people performing the same work in the same industries. Higher salary range should be paid by an employer should the PWDs contribute more ideas towards improving existing products and services ( Malcolm Pace Debono, 2018).

### Career advancement

Career advancement is another critical component in cultivating PWDs’ innovation management involvement. It can be used to recognize the value and potential of the candidates ( Meeks *et al*., 2019). Career advancement and growth depend on the willingness of PWDs to learn and develop new skills and techniques to enhance the existing product and service ( Nautiyal, 2021). PWDs will be more likely to continue innovating products and services if they are given more chances for job promotion and training ( Suresh & Dyaram, 2020).

### Compensation

Compensation is another main motivation for PWDs to become innovative in their work ( Park & Park, 2019). PWDs will feel more psychologically secure and contribute new ideas if the current job provides convincing protection to their life, creativity and knowledge ( Ta *et al*., 2021). Compensation can be a useful and powerful tool that affects PWDs in an organization because company recognition and protection for occupation related loss, suffering, or injury will motivate employees to contribute more ideas to improve the current performance of the company ( Meeks *et al*., 2019).

## Methods

**Figure 1.**
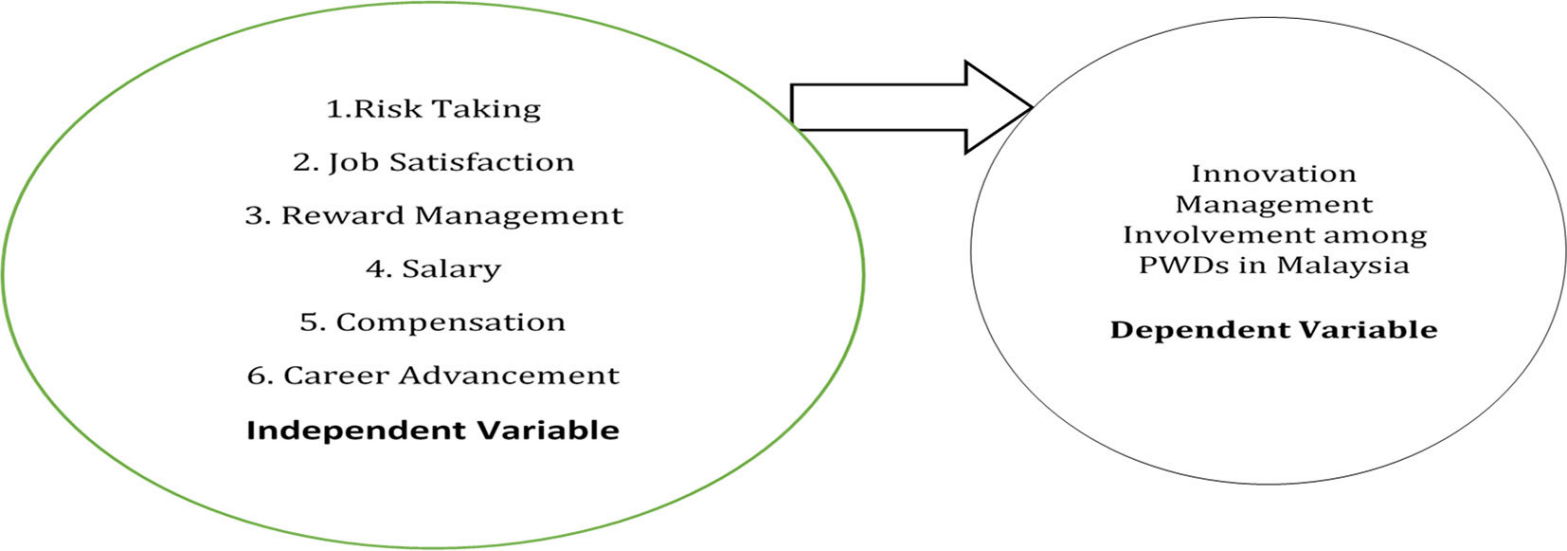
Research framework.

The following research framework was developed to determine the key determinant of innovation management among PWDs in Malaysia. Figure 1 shows the research model.

### Study design

This study is a quantitative survey of involvement in innovation management among persons with disabilities in Malaysia. The data collection period was four months, from 1 January 2021 to 30 April 2021. The survey was conducted on a sample of 200 respondents in SMEs in big cities in Malaysia, such as Kuala Lumpur, Selangor, Johor and Penang. These locations were selected as they are the fastest-growing big cities in the country, and have the highest number of PWD employees, which would allow the desired respondents to be reached.

### Data collection

The survey was created by the researchers using a software called SurveyLab. A copy of the survey used can be found under *Extended data* ( Yuen, 2021). The data collection involved conducting face-to-face recruitment, exposure and data collection with the respondents from 1 January 2021 to 30 April 2021. To ensure the respondents had adequate knowledge in innovation management, the targeted respondents for this study were observed from afar to ensure that they were involved in innovation management activities at their respective workplace in SMEs. Then, the researcher approached the respondents and invited them to participate in this study. Written consent was sought from participants prior to conducting the survey.

Since the target respondents are PWDs, the researchers accompanied each respondent to provide immediate assistance while they were completing the paper survey. Respondents were given 30 minutes to complete the survey. Responses were collected from the respondents immediately after they answered to ensure that a sufficient sample size could be arrived at. Within a four-month period (1 January 2021 to 30 April 2021), 200 valid responses were gathered and used for further analysis. A 5-point Likert scale 1-strongly disagree, 2-disagree, 3-neutral, 4-agree and 5-strongly agree was used in the survey to measure participants’ responses to risk-taking, job satisfaction, reward management, salary, compensation, career advancement and innovation management involvement

Pre-testing validation was conducted face-to-face to ensure that respondents fulfilled three pre-testing validation criteria before they were allowed to answer the survey. The three validation criteria were 1. Respondent must be a current PWD employee in a SME, 2. Respondent must have at least one year of working experience 3. Respondent must have adequate knowledge in innovation management. SMEs that hire PWD employees were identified from https://www.talentcorp.com.my, an official government website for talent management in Malaysia.

After the pre-testing validation, pilot testing was conducted. Pilot testing was conducted among 50 respondents in SMEs before the actual study to test the correctness of the survey instructions and ensure that all the respondents would be able to follow the directions as indicated. Preliminary pilot testing also provides better information on whether the survey is effective in fulfilling the purpose of the study. A grammatical correction was made after pilot testing to ensure the clarity of the survey instruction.

Reliability testing for internal consistency reliability was then conducted. The Cronbach's Alpha reliability coefficient was computed. Survey items measuring risk-taking, job satisfaction, reward management, salary, compensation, career advancement and innovation management involvement have high Cronbach's Alpha coefficients ranged from.72 to.84, All survey items yielded satisfactory internal consistency.

### Sampling

In this research, purposive sampling was used to recruit 200 suitable PWD respondents from SMEs in Malaysia. Purposive sampling refers to selecting target respondents based on characteristics of a population and the objective of the study.

### Analysis

This study used statistical software package, IBM SPSS Statistics version 23.0 to process and analyze the data, IBM SPSS Statistics Version 23.0 was employed for data screening for Common Method Variance (CMV) to eliminate bias caused by the variations in responses to the survey instrument. Data screening results indicated that this survey was free from the CMV bias threat. IBM SPSS Statistics Version 23.0 was also utilized to perform Multiple Linear Regression Analysis to test the effects of risk-taking, job satisfaction, reward management, salary, compensation, and career advancement on the innovation management involvement of PWDs. The R-square value of the Multiple Linear Regression Analysis is a statistical measure used to determine how close the data are to the fitted regression. The higher the R-squared, the better the model.

### Ethics statement

Ethical approval was obtained for this project from the Research Ethics Committee (REC) Multimedia University (Ethical Approval Number: EA1312021). Written consent was obtained from participants for the use of and publication of their data. A written consent statement was printed on the survey. Respondents were required to tick the written consent form before they started the survey.

## Results

200 respondents completed the survey. As shown in Table 1, male respondents represented 54.9% or 110 out of the total 200 respondents. While the number of female respondents represented 44.1% or 90 out of the total 200 respondents. Seventy-nine percent or 158 respondents had one-five years working experience and 21.0% of them had more than five years working experience. Sixty-three percent or 127 respondents are active in innovation management while 28.5% or 57 of them are very active in innovation management. The dataset for this study can be found in the *Underlying data* ( Yuen, 2021).

**Table 1.** Participant demographics.

	Frequency	Percentage
Gender		
Male	110	54.9
Female	90	44.1
Working experience		
1-5 years	158	79,0
>5 years	42	21,0
Innovation management involvement		
Very active	57	28,5
Active	127	63.5
Less active	16	8.0

IBM SPSS Statistics Version 23.0 was utilized to perform Multiple Linear Regression Analysis to test the effect of risk-taking, job satisfaction, reward management, salary, compensation, and career advancement on the innovation management involvement of PWDs. The R-square value of the Multiple Linear Regression Analysis is a statistical measure of how close the data are to the fitted regression. The higher the R-squared, the better the model. Based on the results of Multiple Linear Regression analysis in Table 2, the R-square value is at 0.645. This indicates that 64.5% of the innovation management involvement is explained by independent variables. It implies that the four significant independent variables (salary, compensation, career advancement and reward management) contribute approximately 64.5% toward the dependent variable (innovation management among PWDs in Malaysia) at the significance of <0.005 level. Career advancement is the most important factor affecting innovation management (Beta coefficient = 0.308), followed by reward (Beta coefficient = 0.188), salary (Beta coefficient = 0.150) and compensation (Beta coefficient = 0.139),

**Table 2.** Multiple linear regression results.

Factor	Beta coefficient	Significance	R square
(Constant)	0.739	0.000	0.645
Salary	0.150	0.018	
Risk-taking	0.004	0.072	
Compensation	0.139	0.048	
Career advancement	0.308	0.000	
Reward	0.188	0.035	

## Discussion

Out of 200 PWDs surveyed in this study, 178 claimed that they are no longer interested in innovation management and might leave the organization within two years. Career advancement is the most important factor affecting PWDs’ innovation management involvement in their organization. This is because career advancement is part of lifelong learning that can create an opportunity for the PWDs to innovate products and services. Career advancement can ensure that PWDs have related qualifications, experience and the ability to deal with product and service innovation, career advancement can unleash PWDs’ potential knowledge and skills in order to fulfill innovation requirements.

Salary is a crucial financial incentive to enhance innovation involvement among PWDs. If the company consistently provides a reasonable salary for their PWDs, PWDs will be more motivated to engage in innovation management. SMEs have to update their salary for knowledge workers to make sure that it is on par with counterparts. Moreover, reward is also significant in PWD retention. Appropriate rewards and recognition from company supervisors encourage PWDs to contribute their innovative effort towards jobs. SMEs can express their gratitude through email, gifts, company trips or through offering extra leave. Rewards could be based on monthly performance to motivate PWDs to innovate more products and services. Compensation is another main motivator for PWDs to enhance their innovation involvement. Adequate medical pay, medical facilities, transportation services, additional leave and pension welfare are critical to make PWDs feel valued by the organization. Risk-taking is not a crucial motivator for PWDs because, in SMEs, product and service innovation risks are borne by the owners or senior managers and not the employees.

These four factors are crucial for SMEs in Malaysia to improve PWDs’ involvement in innovation management within the organization. As innovation management is essential for the sustainability of the business in SMEs, this research will help business operators to better retain talented PWDs in innovating high-quality products and services.

SMEs in Malaysia can use the findings of this research as a reference to improve company weaknesses and encourage more PWDs to get involved in innovation management in the next decade. The use of a cross-sectional sample limits the explanation of the hypothesized relationships. The relatively small initial sample (200 respondents) for survey distribution resulted in low generalizability of the data. Further research could use a longitudinal sample with a larger sample size, which would allow the study of relationships of causality, offering a more comprehensive view of the effect of factors on PWDs and innovation management over the long term.

## Data availability

### Underlying data

DANS EASY: Innovation management among persons with disability in Malaysia

https://doi.org/10.17026/dans-x4t-vnz5 ( Yuen, 2021)

This project contains the following underlying data:

• F1000Datasets+Codebook.xlsx (Survey data.)

### Extended data

DANS EASY: Innovation management among persons with disability in Malaysia

https://doi.org/10.17026/dans-x4t-vnz5 ( Yuen, 2021)

This project contains the following extended data:

• Questionnaire on Innovation Management among Persons with Disability in Malaysia.pdf

Data are available under the terms of the Creative Commons Zero “No rights reserved” data waiver (CC0 1.0 Public domain dedication).
