# Supplementary material for: Innovation management involvement among persons with disabilities in Malaysia
Source: F1000Res. 2022 Sep 6;11:180. Originally published 2022 Feb 14. [Version 2] doi: 10.12688/f1000research.74202.2 (PMC9453107; doi:10.12688/f1000research.74202.2)
Supplement: Peer review history

# Peer review: Reviewer response for version 2

**DOI:** 10.5256/f1000research.137826.r149543

**Published:** 2022-09-07

**Authors:** Kuok Ho Daniel Tang

**Affiliations:** 1 Department of Environmental Science, University of Arizona at Northwest A&F University, Yangling, Shaaxi Province, China

The paper has shown substantial improvement and the authors are lauded for the effort put into making the improvement. There are no further comments.

Is the work clearly and accurately presented and does it cite the current literature?

Partly

If applicable, is the statistical analysis and its interpretation appropriate?

Partly

Are all the source data underlying the results available to ensure full reproducibility?

Yes

Is the study design appropriate and is the work technically sound?

Yes

Are the conclusions drawn adequately supported by the results?

No

Are sufficient details of methods and analysis provided to allow replication by others?

Yes

Reviewer Expertise:

Occupational safety and health, climate change, environmental management

I confirm that I have read this submission and believe that I have an appropriate level of expertise to confirm that it is of an acceptable scientific standard.

# Peer review: Reviewer response for version 2

**DOI:** 10.5256/f1000research.137826.r149542

**Published:** 2022-09-06

**Authors:** Yuen-Onn Choong

**Affiliations:** 1 Faculty of Business and Finance, Universiti Tunku Abdul Rahman, Kampar, Malaysia

I am happy to see the improved version of manuscript and all comments are well addressed. Thus, I have no further comments.

Is the work clearly and accurately presented and does it cite the current literature?

Yes

If applicable, is the statistical analysis and its interpretation appropriate?

Yes

Are all the source data underlying the results available to ensure full reproducibility?

Yes

Is the study design appropriate and is the work technically sound?

Yes

Are the conclusions drawn adequately supported by the results?

No

Are sufficient details of methods and analysis provided to allow replication by others?

Partly

Reviewer Expertise:

My area of research is organisational behaviour, educational management, tourism management and SME studies.

I confirm that I have read this submission and believe that I have an appropriate level of expertise to confirm that it is of an acceptable scientific standard.

# Peer review: Reviewer response for version 1

**DOI:** 10.5256/f1000research.77929.r140817

**Published:** 2022-06-20

**Authors:** Kuok Ho Daniel Tang

**Affiliations:** 1 Department of Environmental Science, University of Arizona at Northwest A&F University, Yangling, Shaaxi Province, China

Title

- It suffices to name the paper “Innovation management among persons with disabilities in Malaysia”. Survey is not necessary as it indicates the method. Perhaps the authors may consider refining “innovation management” to capture the contents of the paper more precisely.

Abstract

- The authors may consider providing a summary of the analysis involved, as part of the methods.

Introduction

- The authors may wish to explain why a lack of participation of PWDs in innovation management could hinder innovation life cycle, particularly if an organization does not employ PWDs or if the existing policy and environment deters the involvement of PWDs. Innovation life cycle could also be contributed by individuals other than PWDs. The authors may consider arguing on the integration of PWDs in the workforce, hence innovation.
- The authors claim that many organizations in Malaysia fail to meet the job-related needs of PWDs. Are PWDs more demanding than other segments of the society in work? Or it is because organizations are simply not prepared to employ PWDs.

Literature review

- The literature review here focuses on the constructs of the study. Yet, there is no mention of the theoretical framework adopted from which these constructs have been derived. Therefore, the constructs may appear random.
- The literature review should also state the crucial work previously conducted in this area and the knowledge gaps this study aims to fill in

Methods

- As with other survey-based studies, the authors may wish to explain on the sampling adequacy.
- The authors may want to specify what innovation management activities would qualify the PWDs to be the participants of this study.

Results

- The authors may want to present the descriptive statistics of the survey as well.
- It may be useful to perform a correlational analysis of the constructs for this cross-sectional study.

Discussion

- The authors may compare the findings of this study to those of other relevant studies.
- References could be provided for the inferences given in the discussion.

General

- The authors may add a concluding remark which reiterates the significant findings as well as the significance and specific practical implications of the findings.

Is the work clearly and accurately presented and does it cite the current literature?

Partly

If applicable, is the statistical analysis and its interpretation appropriate?

Partly

Are all the source data underlying the results available to ensure full reproducibility?

Yes

Is the study design appropriate and is the work technically sound?

Yes

Are the conclusions drawn adequately supported by the results?

No

Are sufficient details of methods and analysis provided to allow replication by others?

Yes

Reviewer Expertise:

Occupational safety and health, climate change, environmental management

I confirm that I have read this submission and believe that I have an appropriate level of expertise to confirm that it is of an acceptable scientific standard, however I have significant reservations, as outlined above.

# Peer review: Reviewer response for version 1

**DOI:** 10.5256/f1000research.77929.r138663

**Published:** 2022-05-30

**Authors:** Yuen-Onn Choong

**Affiliations:** 1 Faculty of Business and Finance, Universiti Tunku Abdul Rahman, Kampar, Malaysia

The authors should relate the issue to innovation management. The issue is focused on turnover intention or retention of PWCs. However, the explanation of the linkage between the issue and innovation management is weak and limited in the current form. Are there any similar past studies which can be included and explained so the authors could show how these relevant studies differ as compared to your study?

 For the methodology :

 What sampling technique was used in this study and why? Next question is how do you ensure the generalizability and representativeness of the sample toward the targeted population? It is well understood that there are three selection criteria. However, how do the author assess respondent innovation management? 

 Are there any theoretical implication for this study? 

 The work should have managerial implications. I would suggest author to provide implications based on the current usual practices and policies. What effective interventions can be offered to all these stakeholders?

 There should be a standalone section for conclusions. I would like the authors to talk about the key findings and what key implications can be recapped in the conclusion.

Is the work clearly and accurately presented and does it cite the current literature?

Yes

If applicable, is the statistical analysis and its interpretation appropriate?

Yes

Are all the source data underlying the results available to ensure full reproducibility?

Yes

Is the study design appropriate and is the work technically sound?

Yes

Are the conclusions drawn adequately supported by the results?

No

Are sufficient details of methods and analysis provided to allow replication by others?

Partly

Reviewer Expertise:

My area of research is organisational behaviour, educational management, tourism management and SME studies.

I confirm that I have read this submission and believe that I have an appropriate level of expertise to confirm that it is of an acceptable scientific standard, however I have significant reservations, as outlined above.
